# Sclerosing Mesenteritis Managed Conservatively With Prednisone

**DOI:** 10.7759/cureus.35419

**Published:** 2023-02-24

**Authors:** Saipriya Gande, Natalie N Nguyen, Thor S Stead, Rohan Mangal, Latha Ganti

**Affiliations:** 1 Medicine, Academy at the Lakes, Lutz, USA; 2 Biology and Medicine, Brown University, Providence, USA; 3 Medicine, The Warren Alpert Medical School of Brown University, Providence, USA; 4 Medicine, Johns Hopkins University, Baltimore, USA; 5 Medicine, University of Miami Miller School of Medicine, Miami, USA; 6 Emergency Medicine, HCA Florida Ocala Hospital, Ocala, USA; 7 Emergency Medicine, Envision Physician Services, Plantation, USA; 8 Emergency Medicine, University of Central Florida College of Medicine, Orlando, USA

**Keywords:** chatgpt, retractile mesenteritis, mesenteric lipodystrophy, sclerosing mesenteritis, mesenteric scleritis, mesenteric panniculitis

## Abstract

The authors present the case of a middle-aged lady with two weeks of abdominal pain. Computed tomography imaging revealed sclerosing mesenteritis. Sclerosing mesenteritis is also known as mesenteric panniculitis and is a chronic fibrosing inflammatory disease that primarily affects the adipose tissue of the mesentery in the small intestine and colon. The clinical presentation, imaging findings, differential diagnosis, and therapeutic management are presented in this report. In our patient’s case, she was able to be managed conservatively, without the need for surgery. This reflects the most benign and self-limiting natural history of the disease.

## Introduction

Sclerosing mesenteritis (SM), also known as retractile mesenteritis, mesenteric lipodystrophy, or mesenteric panniculitis, [1] is a rare inflammatory fibrotic disease of the small intestinal mesenteric fat [2]. The most common symptoms of SM include nausea, bloating, and abdominal pain [2]. However, because the symptoms are quite general, imaging and histological markers can be helpful in diagnosing SM. SM can also be identified by a “fat ring sign” or “tumoral pseudocapsule sign” in radiology [3]. Elevated IgG4 has also been documented as a histological marker in IgG-related SM [4]. This disease is more common among Caucasians and men, with a male-to-female ratio of 2.3:1 [5]. It tends to affect people during their fifth to seventh decades of life, with a mean age of 55+- 19.2 years of age [2,5]. A systematic review of 192 cases revealed several possible risk factors for developing the condition. One study found that 28.6% of patients had a history of prior surgery or abdominal trauma, 8.9% had a history of malignancy, and 5.7% had a history of autoimmune disease [5]. First-line treatment typically involves a combination of tamoxifen and corticosteroid or corticosteroid alone, but in patients with persistent obstruction, surgical intervention may be required [6].

## Case presentation

**Figure 1:**
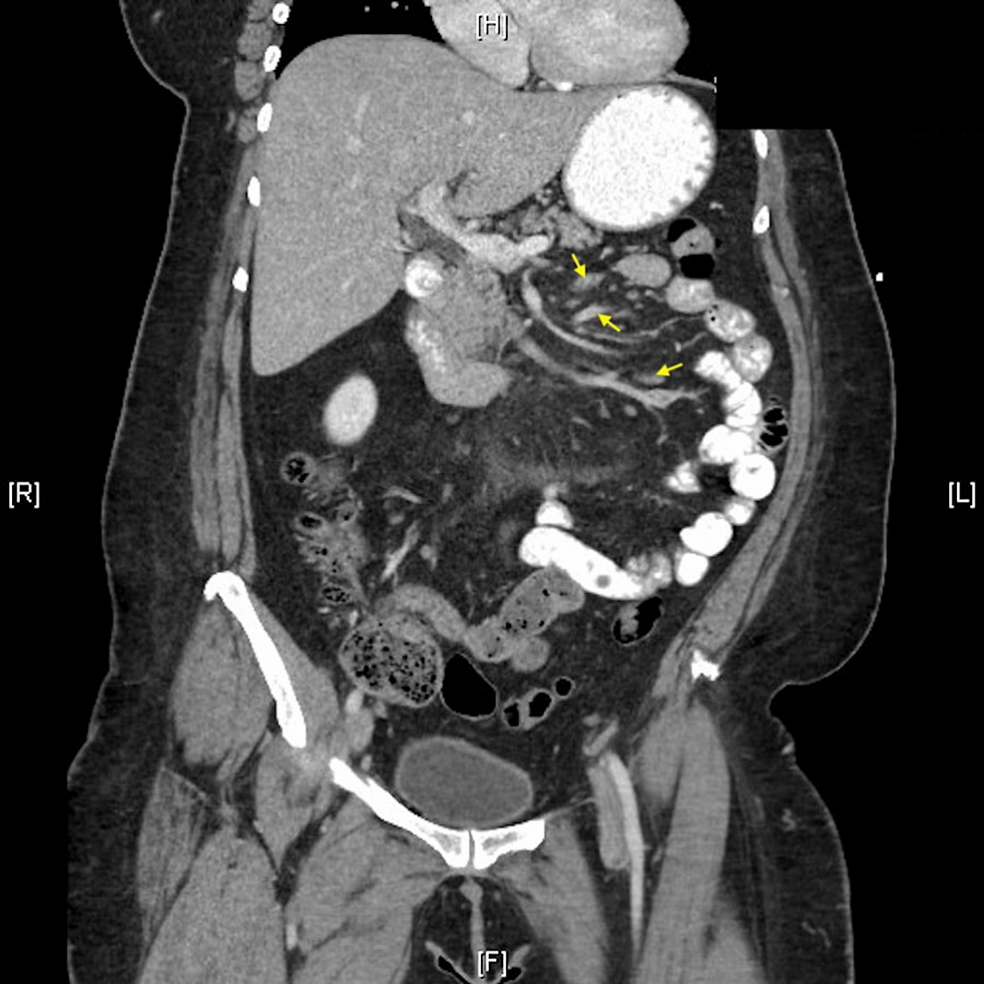
Axial abdominal contrast CT demonstrating mesenteric fat stranding (arrows)

**Figure 2:**
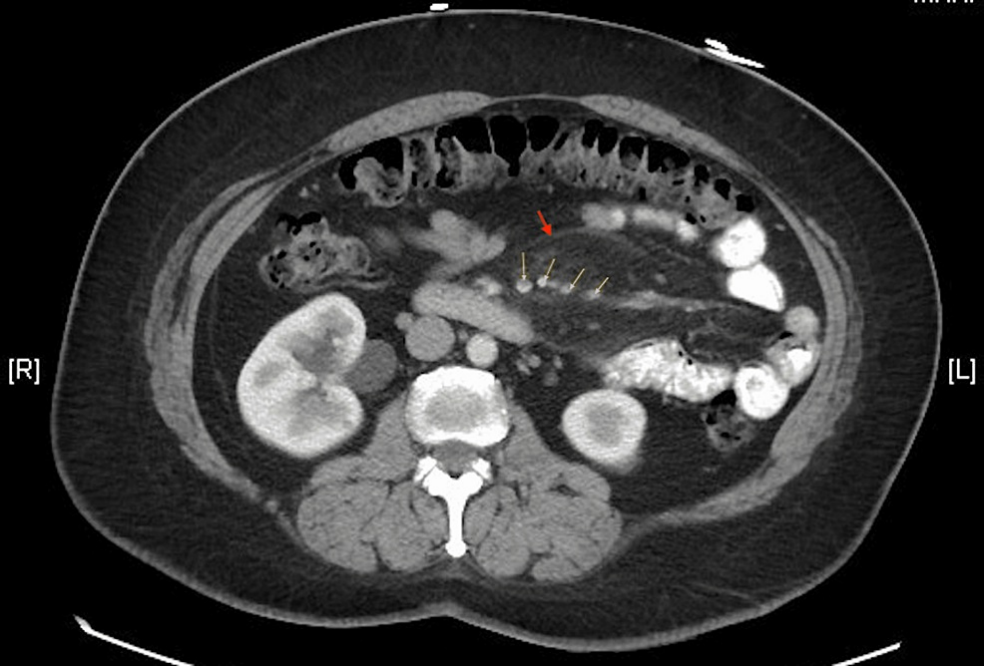
Coronal abdominal CT with contrast demonstrating well-defined mass in mesentery with a pseudocapsule (red arrow). Contained within is focally enhancing mesenteric fat (yellow arrows)

A 57-year-old female presented to the ED with two weeks of left-sided flank pain. She described the pain as sharp and radiating to her left groin. She stated the pain was constant with no modifying factors. The patient denied any nausea, vomiting, dysuria, frequency, hematuria, diarrhea, fever, chest pain, difficulty breathing, or bloody stools. Her past medical history was significant for asthma for which she used an albuterol inhaler, hypertension for which she took lisinopril, and diabetes for which she took insulin. Her past surgical history was significant for cholecystectomy. She is a nonsmoker. She had no known drug allergies. Her vital signs revealed a temperature of 97.4° F, blood pressure 178/92 mmHg, pulse of 95 beats per minute, respiratory rate of 18 breaths per minute, and saturating at 99% on room air. On physical examination, the patient was a middle-aged female with mild to moderate discomfort secondary to abdominal pain. Examination of the abdomen itself was soft, and mildly tender in the left lower quadrant, without rebound or guarding. Laboratory analysis was notable for mild leukocytosis (Table 1). Computed tomography (CT) scans demonstrated a mesenteric root panniculitis (sclerosing mesenteritis) of approximately 10 x 7 x 6 cm. Also noted is diverticulosis (Figures 1, 2).

General surgery was consulted and ordered a contrast CT which re-demonstrated mesenteric panniculitis, without bowel obstruction, colonic dilatation or wall thickening. The patient was admitted for observation. There were no signs of acute abdomen on serial exams which corroborated no bowel wall thickening on CT scans. Besides a mild leukocytosis, the patient had no signs of sepsis. She was managed with prednisone 40mg daily and normal saline at 125mL/hr. She had an uneventful discharge home on hospital day 2.

## Discussion

The diagnosis of sclerosing mesenteritis can be challenging, as the condition is rare, and symptoms can be similar to other abdominal disorders. The process typically involves a combination of physical examination, imaging tests, and biopsy. Physical examination may reveal signs of abdominal discomfort or pain, dyspepsia, as well as an enlarged or tender abdomen [7].

Radiologic findings in sclerosing mesenteritis can vary depending on the stage of the disease and the imaging modality used. Computed tomography (CT) scans are considered the most sensitive and specific imaging modality for the diagnosis of sclerosing mesenteritis. On CT, sclerosing mesenteritis typically appears as thickening and enhancement of the mesentery, with a characteristic “coffee bean” or “target” sign, which is a combination of a central area of low attenuation and a peripheral ring of enhancement. The mesenteric fat may appear hyperdense. There is also often a well-defined mass in the mesentery that typically does not produce a mass effect. The mass is also usually delineated by what is termed a pseudocapsule. Also, the characteristic is small, scattered lymph nodes. Similar CT features are seen in other diseases such as carcinomatosis, carcinoid tumor, lymphoma, desmoid tumor, and mesenteric edema [8].

Magnetic resonance imaging (MRI) can also be used to visualize the thickening of the mesentery, with high-signal intensity on T2-weighted images and enhancement with contrast when the disease is in the inflammatory stage. When fibrosis is predominant, the disease appears as a localized mass of fibrous tissue, hypointense in both T1 and T2 sequences [8]. Ultrasonography or endoscopic ultrasound may show thickening and hyperechogenicity of the mesentery [9], but usually, the diagnosis is suspected after CT or MRI imaging and confirmed by biopsy.

It is important to note that sclerosing mesenteritis can be asymptomatic, and the imaging findings may be subtle, thus the diagnosis may be missed or delayed in some cases. Additionally, the imaging findings can be similar to other abdominal conditions, such as inflammatory bowel disease or lymphoma. A biopsy is considered the gold standard for confirming a diagnosis of sclerosing mesenteritis. The biopsy typically shows fibrosis, lymphocytic infiltration, and/or granuloma formation [10]. However, since biopsy is an invasive technique, it is generally reserved for suspected malignancy.

Treatment for sclerosing mesenteritis typically involves medications to manage symptoms and reduce inflammation. These can include non-steroidal anti-inflammatory drugs (NSAIDs) and immunosuppressive agents. Surgery is reserved for bowel obstruction or perforation or complicated panniculitis refractory to medical treatment [6]. In many cases, where the condition is not severe, conservative treatment with observation would suffice. This was the case with our patient. A similar resolution of sclerosing mesenteritis with steroid therapy is documented in the literature [11,12].

## Conclusions

Sclerosing mesenteritis is a relatively rare condition that can be seen in association with neoplasms, postoperatively, and de novo. It occurs on a spectrum, from an acute inflammatory process to a more chronic fibrosis-related presentation. Diagnosis is usually suspected on the exam and confirmed with CT or MRI imaging followed by a biopsy. Our patient had a relatively benign course which did not require surgical intervention but rather was able to be managed with only steroid therapy.

## Figures and Tables

**Figure 3:**
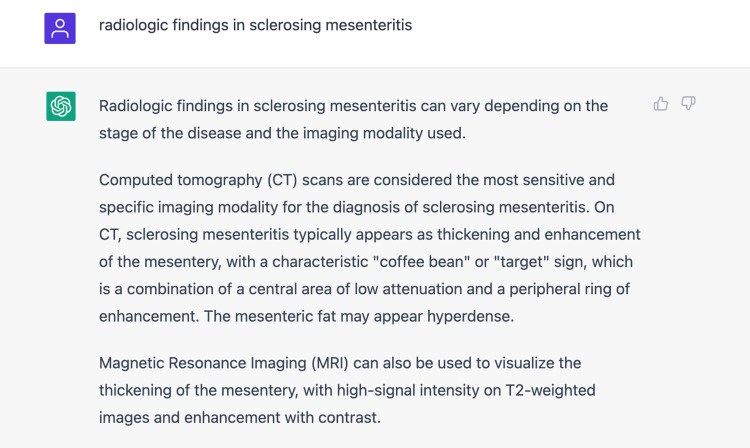
Screenshot of first query in ChatGPT

**Figure 4:**
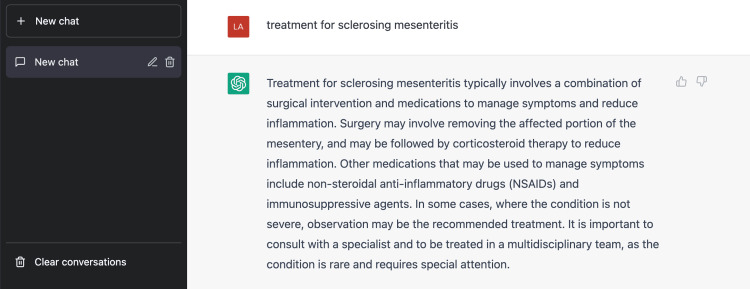
Screenshot of second query in ChatGPT

## Appendices

This case report was submitted as part of the "A call for case reports contest written with the assistance of ChatGPT." As novice users, we gave the ChatGPT software the directed prompts as shown below. We used the generated text to look up references to support these statements in PubMed. We manually retrieved the citations and wrote out the references. We then copy-pasted our entire article in plagiarism detection software (iThenticate®) to ensure the generated text was not plagiarized.
